# Socioeconomic and ethnic inequalities increase the risk of type 2 diabetes: an analysis of NHS health check attendees in Birmingham

**DOI:** 10.3389/fpubh.2024.1477418

**Published:** 2024-11-27

**Authors:** Chung Him Au-Yeung, David Ellis, Alexander Dallaway, Jenny Riley, Justin Varney, Rebecca Howell-Jones

**Affiliations:** ^1^Public Health, Birmingham City Council, Birmingham, United Kingdom; ^2^School of Health and Society, Faculty of Education, Health and Wellbeing, University of Wolverhampton, Wolverhampton, United Kingdom; ^3^Warwickshire Institute for the Study of Diabetes, Endocrinology and Metabolism (WISDEM), University Hospitals Coventry and Warwickshire NHS Trust, Coventry, United Kingdom

**Keywords:** glycated hemoglobin A1c, socioeconomic factors, social determinants of health, health inequities, primary health care, body mass index, logistic models

## Abstract

**Introduction:**

Birmingham has a significantly higher type-II diabetes prevalence than the national average. This study aimed to investigate the association of socioeconomic deprivation and ethnicity on the risk of diabetes in Birmingham.

**Methods:**

Data were included from 108,514 NHS Health Checks conducted in Birmingham between 2018 and 2023. Attributable fraction and multinomial logistic regression were used to estimate the number of events avoidable and the prevalence odds ratios (POR) of determinants respectively.

**Results:**

Attributable fraction analysis estimated that 64% of diabetes and 44% of pre-diabetes cases could be attributed to socioeconomic deprivation. Specifically, if Asian attendees in the least deprived areas had the same risk as White individuals in the least deprived areas, there would have been 1,056 fewer cases of diabetes and 2,226 fewer cases of pre-diabetes. Diabetes was significantly associated with Asian ethnicity (POR = 5.43, *p* < 0.001), Black ethnicity (POR = 3.15, *p* < 0.001) and Mixed ethnicity (POR = 2.79, *p* < 0.001). Pre-diabetes was also significantly associated with Asian ethnicity (POR = 3.06, *p* < 0.001), Black ethnicity (POR = 2.70, *p* < 0.001) and Mixed ethnicity (POR = 2.21, *p* < 0.001). The interaction effects between ethnicity and deprivation posed a greater risk of diabetes, especially for Asian attendees in the first (POR = 9.34, *p* < 0.001) and second (POR = 6.24, *p* < 0.001) most deprived quintiles.

**Discussion:**

The present findings demonstrate the association of ethnicity and socioeconomic deprivation on the risk of diabetes and pre-diabetes. It underscores the necessity for targeted interventions and policies to address these inequalities.

## 1 Introduction

In the UK, Type 2 diabetes represents ~90% of all diabetes cases among adults (1). There are currently 4.4 million people living with Type 2 diabetes, with more than 13.6 million people an estimated at increased risk of Type 2 diabetes and other serious health conditions (2). Diabetes presents a substantial public health and socioeconomic burden, with an estimated financial impact of at least £10 billion annually on the National Health Service (NHS), which is ~10% of the entire NHS budget. The healthcare cost of managing Type 2 diabetes is mainly due to complications arising from diabetes (2). These complications can impact on quality of life and impair physical, mental, cognitive, psychological and social status (3).

The NHS Health Check (HC) programme is a preventive free health check-up in England. NHS HCs were introduced in 2009 with the aim to identify and manage individuals at risk of cardiovascular disease or other conditions including diabetes. Individuals who aged between 40 and 74 with no pre-existing cardiovascular conditions (e.g. heart disease or stroke) or certain risk factors for these conditions are eligible for HCs every five years (4). NHS HC programme within Birmingham is one of the mandated public health services commissioned by Birmingham City Council. The estimated diabetes prevalence among adults aged 17 and above in Birmingham has risen steadily from 6.6% in 2009/10 to 9.1% in 2021/22, consistently exceeds the national prevalence in England (5.4%–7.3% during the same period) (5). Given the aging population in the UK, owing to advances in healthcare and other lifestyle factors, aging has become a growing public health concern. The percentage of patients registered with Type 2 diabetes among people aged 40–64 years is 43.9% in 2021/22 period and this has increased from 41% since 2015/2016 period (6). Older age is a key risk factor for diabetes and pre-diabetes (7, 8) and older adults are more likely to develop cardiovascular, retinal and renal comorbid complications (9, 10). Changes in socio-demographic and clinical characteristics as a function of age further emphasizes the importance of it as a moderating variable for diabetes and associated outcomes (11).

The influence of wider determinants on health outcomes and inequalities is well known. The Marmot reviews demonstrated the close links between health inequalities and social determinants and the impact on health outcomes including diabetes (12, 13). Birmingham faces high levels of deprivation and diversity within its ethnic communities, leading to marginalized groups due to structural barriers like poverty, education, and housing (14). These barriers pose difficulties in accessing affordable, healthy food and in participating in regular physical activity. These challenges serve to worsen the quality of life and health outcomes, thus exacerbating health inequalities (15). Despite acknowledging the necessity to address wider determinants, efforts to address the root social causes of ill health and reduce inequality have not been consistently undertaken. A 2014 literature review highlighted that the majority of the US-based studies investigating the social determinants of diabetes have predominantly employed descriptive analysis (16). The authors stressed the limitation of these studies, particularly due to the small sample size.

A UK study in 2017 by Chizia and Bellingham-Young investigated a set of social determinants for Type 2 diabetes to provide insights for population-based intervention that addresses social and economic inequalities (17). However, this study is limited by its use of group-level data. This approach could potentially reduce the precision of estimates and limit the ability to detect relationships between determinants and outcomes. Furthermore, casual inferences could be incorrectly drawn due to Simpson's paradox which may misguide public health and policy (18).

Several studies have primarily concentrated on investigating the social determinants of Type 2 diabetes in children and young people (19, 20). These studies have specifically examined differences in risk for Type 2 diabetes between ethnic groups. However, there is limited focus on middle-aged and older adults despite their increased risk.

Data on social determinants are not always available or poorly recorded in electronic health records (21, 22). As a result, studies have typically used deprivation indices as a proxy variable within their research purposes in different academic areas (23–25). Furthermore, there is very limited UK-based research that investigates the impact of deprivation and ethnicity in a multifaceted approach on diabetes and these studies adopt descriptive statistics (26, 27). Only one recent UK study in 2023 by Nagar and colleagues employed inferential statistics and interaction effect to investigate this relationship but possible confounders are not controlled (28).

The aim of this study was to investigate the impact of socioeconomic deprivation and ethnicity and their interaction with Type 2 diabetes among middle-aged and older adults in Birmingham.

## 2 Methods

Data were obtained from NHS HC completed between 22/06/2018 and 30/06/2023. These data were provided by the Midlands and Lancashire Clinical Support Unit (MLCSU) who extracted the General Practice (GP) data from Egton Medical Information Systems (EMIS) and The Phoenix Partnership (TPP) System One.

### 2.1 Data cleaning

Outliers in the data and records with entry or measurement errors were removed from the dataset. *Z*-score calculations were utilized for key variables: height, weight, and HbA1c and data that were greater than three standard deviations away from the mean were removed. A total of 2,748 HC attendees with missing HbA1c values were excluded from the data set. The data cleaning process led to a reduction in the data set from 108,514 to 91,711 observations through list-wise deletion.

### 2.2 Definition of outcomes

A dependent variable with three categories was used in this study, namely “Non-diabetes,” “diabetes,” and “Pre-diabetes.” The diagnosis of these conditions was not explicitly given in the data. Instead, the HbA1c level is used to ascertain whether the HC attendees fell into the diabetic or pre-diabetic range. The World Health Organisation's (29) guideline for the diagnosis of Type 2 diabetes by HbA1c has been used in this paper, with HbA1c equal to or above 48 mmol/mol classified as diabetes, HbA1c between 42 mmol/mol and 47 mmol/mol classified as pre-diabetes and HbA1c below 42 mmol/mol classified as non-diabetes.

### 2.3 Definition of socioeconomic deprivation

Index of Multiple Deprivation (IMD) deciles at the lower super output area (LSOA) of subject's GP practices were given in the dataset. The 20% most deprived areas in England were grouped into “IMD quintile 1,” the 20%–40% IMD decile was grouped into “IMD quintile 2” and the least deprived 60% IMD decile was grouped into “IMD quintile 3+.” “IMD quintile 3+” is the reference category.

### 2.4 Explanatory variables

A total of 188 unique ethnicity classes were recorded but these classes were inconsistent and overlapping between different ethnicities. Therefore, this study followed the Office for National Statistics categorization system from the UK census and stratified these descriptions into broader ethnic groups: “Asian,” “Black,” “Mixed,” “Other,” and “White.” The “White” cohort is the reference category.

Explanatory variables in the dataset included age, gender, body mass index (BMI), diastolic blood pressure, systolic blood pressure, smoking status, alcohol units, and levels of physical activity. Detailed methodological information on coding these factors as the explanatory variables in the analyses is described in the second section of the Supplementary material.

### 2.5 Interaction effects

This study used the interaction between IMD quintile (three levels) and ethnicity (five levels), resulting in 15 combinations of these two categories. The interaction was modeled in a separate regression model and controlled for all other explanatory variables. The interaction effect was reported as the overall pattern which comprehensively encapsulates both the main effects and interaction effects, collectively referred to as the “net effect” (30). The interaction effect's statistical significance was based on the standard error of the net effect, derived as the square root of the variance of the sum (31, 32). The detailed calculation can be found in the fourth section of the Supplementary material.

### 2.6 Handling of missing data

The proportion of missing values varied from 0 to 49%. Only 33% of observations represented complete cases with no missing item (Supplementary Figure S3 details the distribution and missing pattern). Little's Missing Completely at Random test (Supplementary Table S4) indicated that missing values were not missing completely at random (33). Therefore, this study employed the Multiple imputation with chained equations (MICE) to handle the missing values in the data set. The imputation of missing ethnicity data was enhanced by joining information on the ethnic distribution of patients registered to each GP practice. Since the distribution data was a type of composition data, the additive log-ratio transformation was applied (34).

The number of imputed data sets chosen was 50, which aligns with the guidance of one imputed data set for every 1% of incomplete cases (35–37). The means and standard errors were calculated through a process of pooling using Rubin's rules (38) for the adjusted attributable fractions and multinomial logistic regression. The unadjusted attributable fractions were calculated using data with complete case only. The results of the unadjusted attributable fractions and multinomial logistic regression (available in Supplementary Table S5, Supplementary Figure S5, and Supplementary Table S6 respectively) with complete case analysis were comparable to the estimates based on the multiple imputation. However, the utilization of imputed data improved the statistical efficiency and effectively mitigated biases.

### 2.7 Statistical analysis

The attributable fraction (AF) in a cross-sectional design represents the proportion of prevalent cases of disease that can be attributed to the exposure (39). In cross-sectional studies, Relative Risk (RR) cannot be directly obtained, so the prevalence ratio (PR) which shares the same mathematical formula but does not imply causality, is commonly used (40). Therefore, the unadjusted AFs of diabetes and pre-diabetes attributed to patients' ethnicity and socioeconomic deprivation were estimated using


(1) $$\begin{array}{l} AF=\frac{PR-1}{PR}, \end{array}$$

where *PR* is the prevalence of the exposed group compared to the unexposed group given by


(2) $$\begin{array}{l} PR=\frac{\text{Prevalence of disease in the exposed}}{\text{Prevalence of disease in the unexposed}}, \end{array}$$

This gives the proportion of negative events that would have been avoided if exposure to risk factors were diminished to the reference level. The “Other” ethnicity was excluded from the calculation due to the limited number of occurrences.

The adjusted AFs were calculated using the multinomial logistic regression. The predicted probability of disease for each individual was generated, and the number of cases was then calculated, which is equivalent to the above formula (41). The variance for the adjusted AFs was derived using the asymptotic variance formula (42). This variance was then applied to the logarithmically transformed AFs to normalize the distribution and stabilize the variance, allowing for the calculation of confidence intervals (43).

Multinomial logistic regression (MLR) was employed to estimate the probabilities associated with the classification of categories within a dependent variable. The use of MLR is justified because the dependent variable has more than two categories, each being nominal and mutually exclusive in nature. The model can be represented by the equation below


(3) $$\begin{array}{l} \ln\;\left(\frac{P\left(\text{Outcome}=j\right)}{P\left(\text{Outcome}=J_{\text{base}}\right)}\right)=a_{j}+\overset{n}{\underset{i=1}{\sum }}\beta_{ji}x_{i},\;j=1,\ldots ,J-1 \end{array}$$

in which β_*ji*_ is a vector of coefficients corresponding to the *i*-th explanatory variable in the *x*_*i*_ vector, with *j* = 1, …, *J*−1; *n* is the number of explanatory variables correspond to each *x*_*i*_; *a*_*j*_ represents the constant associated with the *j*-th outcome categories; *J*_base_ is the reference category (i.e., non-diabetes); *j* represents the outcome categories, which comprises two values (i.e., diabetes and pre-diabetes) relative to the omitted reference category. In contrast to fitting *j* binary logistic regression separately, MLR estimates model parameters with smaller standard errors and greater parsimony when modeling multiple categorical responses simultaneously (44–46). In cross-sectional studies, the odds ratio obtained from the MLR is also referred to as the prevalence odds ratio (POR) (47). Therefore, parameter estimates were reported in POR to ensure consistency.

An alpha level of 0.05 was required for statistical significance in all tests. All data processing was performed in R version 4.3.3. AF was performed in Python 3.13 while MLR and MICE were performed in R version 4.3.3. Data visualizations were performed in both R version 4.3.3 and Python 3.13.

## 3 Results

### 3.1 Descriptive analysis

Table 1 provides an overview of the characteristics of the HC attendees by ethnicity using the non-imputed data set. It can be observed that the prevalence of diabetes and pre-diabetes varies greatly across ethnicity. People of Asian ethnicity had the highest prevalence of diabetes (4.9%), followed by Black (3.2%) and Mixed ethnicity (3.2%), White ethnicity (1.5%) and Other ethnicity (1.1%) respectively. Prevalence of pre-diabetes was found to be highest in Black ethnicity (17%), closely followed by Asian ethnicity (15%), Mixed ethnicity (13%), Other ethnicity (8%) and White ethnicity (5.5%) respectively. Additionally, the prevalence of diabetes and pre-diabetes varies greatly across IMD quintiles. Attendees from IMD quintile 1 had a higher prevalence of diabetes (3.7%) compared to those from IMD quintile 3+ (1.4%). Similarly, attendees from IMD quintile 1 also showed a higher prevalence of pre-diabetes (12%) compared to those from IMD quintile 3+ (6.9%). Further descriptive analysis of the profile of HC attendees is available in the third section of the Supplementary material.

**Table 1:** Main characteristics of the HC attendees in Birmingham between 2018 and 2023.

**Variable**	**Normal^*a*^, *N* = 79,737**	**95% CI**	**Diabetic^*a*^, N = 2,756**	**95% CI**	**Pre-diabetic^*a*^, *N* = 9,218**	**95% CI**
**Gender**						
Female	43,030/48,742 (88%)	88%, 89%	1,246/48,742 (2.6%)	2.4%, 2.7%	4,466/48,742 (9.2%)	8.9%, 9.4%
Male	34,311/40,021 (86%)	85%, 86%	1,374/40,021 (3.4%)	3.3%, 3.6%	4,336/40,021 (11%)	11%, 11%
Missing	2,396		136		416	
**Age category**						
40-54	47,532/53,610 (89%)	88%, 89%	1,308/53,610 (2.4%)	2.3%, 2.6%	4,770/53,610 (8.9%)	8.7%, 9.1%
55-69	27,261/32,155 (85%)	84%, 85%	1,146/32,155 (3.6%)	3.4%, 3.8%	3,748/32,155 (12%)	11%, 12%
70-74	2,445/2,980 (82%)	81%, 83%	149/2,980 (5.0%)	4.3%, 5.9%	386 / 2,980 (13%)	12%, 14%
Missing	2,499		153		314	
**BMI category**						
Normal	24,444/26,398 (93%)	92%, 93%	380 / 26,398 (1.4%)	1.3%, 1.6%	1,574 / 26,398 (6.0%)	5.7%, 6.3%
Underweight	1,229/1,296 (95%)	93%, 96%	8 / 1,296 (0.6%)	0.29%, 1.3%	59 / 1,296 (4.6%)	3.5%, 5.9%
Overweight	30,894/35,147 (88%)	88%, 88%	951 / 35,147 (2.7%)	2.5%, 2.9%	3,302 / 35,147 (9.4%)	9.1%, 9.7%
Obese	22,745/28,382 (80%)	80%, 81%	1,409 / 28,382 (5.0%)	4.7%, 5.2%	4,228 / 28,382 (15%)	14%, 15%
Missing	425		8		55	
**Ethnicity broad**						
White	25,363/27,267 (93%)	93%, 93%	402/27,267 (1.5%)	1.3%, 1.6%	1,502/27,267 (5.5%)	5.2%, 5.8%
Asian	11,307/14,123 (80%)	79%, 81%	690/14,123 (4.9%)	4.5%, 5.3%	2,126/14,123 (15%)	14%, 16%
Black	2,125/2,648 (80%)	79%, 82%	84/2,648 (3.2%)	2.6%, 3.9%	439/2,648 (17%)	15%, 18%
Mixed	2,013/2,393 (84%)	83%, 86%	76/2,393 (3.2%)	2.5%, 4.0%	304/2,393 (13%)	11%, 14%
Other	79/87 (91%)	82%, 96%	1/87 (1.1%)	0.06%, 7.1%	7/87 (8.0%)	3.6%, 16%
Missing	38,850		1,503		4,840	
**Smoking status**						
Never smoked	46,829/54,332 (86%)	86%, 86%	1,783/54,332 (3.3%)	3.1%, 3.4%	5,720/54,332 (11%)	10%, 11%
Current smoker	14,709/16,928 (87%)	86%, 87%	478/16,928 (2.8%)	2.6%, 3.1%	1,741/16,928 (10%)	9.8%, 11%
Ex-smoker	15,882/17,811 (89%)	89%, 90%	424/17,811 (2.4%)	2.2%, 2.6%	1,505/17,811 (8.4%)	8.0%, 8.9%
Non-smoker—history unknown	2,270/2,584 (88%)	87%, 89%	68/2,584 (2.6%)	2.1%, 3.3%	246/2,584 (9.5%)	8.4%, 11%
Missing	47		3		6	
**Broad activity term**						
Moderately physically active	37,129/42,974 (86%)	86%, 87%	1,431/42,974 (3.3%)	3.2%, 3.5%	4,414/42,974 (10%)	10%, 11%
Physically active	20,793/22,901 (91%)	90%, 91%	391/22,901 (1.7%)	1.5%, 1.9%	1,717/22,901 (7.5%)	7.2%, 7.8%
Physically inactive	13,219/15,826 (84%)	83%, 84%	612/15,826 (3.9%)	3.6%, 4.2%	1,995/15,826 (13%)	12%, 13%
Missing	8,596		322		1,092	
**IMD quintile**						
IMD quintile 3+	14,014/15,291 (92%)	91%, 92%	219/15,291 (1.4%)	1.3%, 1.6%	1,058/15,291 (6.9%)	6.5%, 7.3%
IMD quintile 1	53,772/63,413 (85%)	85%, 85%	2,347/63,413 (3.7%)	3.6%, 3.9%	7,294/63,413 (12%)	11%, 12%
IMD quintile 2	11,951/13,007 (92%)	91%, 92%	190/13,007 (1.5%)	1.3%, 1.7%	866/13,007 (6.7%)	6.2%, 7.1%
Missing	0		0		0	
**Alcohol category**						
Higher risk drinking	1,275/1,357 (94%)	93%, 95%	22/1,357 (1.6%)	1.0%, 2.5%	60/1,357 (4.4%)	3.4%, 5.7%
Increasing risk drinking	4,684/4,997 (94%)	93%, 94%	73/4,997 (1.5%)	1.2%, 1.8%	240/4,997 (4.8%)	4.2%, 5.4%
Low risk drinking	20,725/22,495 (92%)	92%, 92%	330/22,495 (1.5%)	1.3%, 1.6%	1,440/22,495 (6.4%)	6.1%, 6.7%
Non-drinker	34,364/41,308 (83%)	83%, 84%	1,633/41,308 (4.0%)	3.8%, 4.1%	5,311/41,308 (13%)	13%, 13%
Missing	18,689		698		2,167	

CI, confidence interval.

^a^Mean (SD) for continuous variables; *n* (Proportion %) for categorical variables.

### 3.2 Attributable fraction

The AFs for diabetes and pre-diabetes attributable to socioeconomic deprivation and ethnicity were calculated and are presented in Table 2. For socioeconomic deprivation, the AF for diabetes was higher in IMD quintile 1 [64%, (64%, 64%)], while the AF for pre-diabetes was lower [44%, (44%, 44%)]. The AF for diabetes was also higher in IMD quintile 2 [58%, (57%, 58%)], while the AF for pre-diabetes was lower [36%, (35%, 37%)].

**Table 2:** The adjusted attributable fractions of diabetes and pre-diabetes which are attributable to deprivation and to ethnicity.

	**Diabetes**		**Pre-diabetes**	
	**Attributable fraction** ^a^	**Excess outcome** ^*a, b*^	**Attributable fraction** ^*a*^	**Excess outcome** ^*a, b*^
**Socioeconomic deprivation**				
IMD quintile 3+	Reference	Reference	Reference	Reference
IMD quintile 1	64.30%	1188	44.02%	2517
	(64.23% to 64.37%)	(1186 to 1189)	(43.86% to 44.18%)	(2508 to 2526)
IMD quintile 2	57.81%	289	35.80%	565
	(57.45% to 58.16%)	(287 to 291)	(35.08% to 36.51%)	(553 to 576)
**Ethnicity**				
White	Reference	Reference	Reference	Reference
Asian	78.20%	1327	60.83%	2812
	(77.98%–78.43%)	(1,323–1,331)	(60.41%–61.25%)	(,2793–2,831)
Black	69.79%	154	61.25%	527
	(68.62%–70.91%)	(152–157)	(60.14%–62.34%)	(517–536)
Mixed	62.50%	101	51.36%	310
	(60.33%–64.54%)	(98–104)	(49.09%–53.54%)	(297–324)

^*a*^Confidence intervals are in parentheses.

^*b*^Excess outcome refers to the number of outcomes in the data set that would have been avoided.

For ethnicity, the AF for diabetes was higher in Asian ethnicity [78%, (78%, 78%)], closely followed by Black [78%, (78%, 78%)] and Mixed ethnicity. The AF for pre-diabetes was higher in Black [61%, (60%, 62%)], followed by Asian [61%, (60%, 62%)] and Mixed ethnicity [51%, (49%, 54%)].

**Figure 1:**
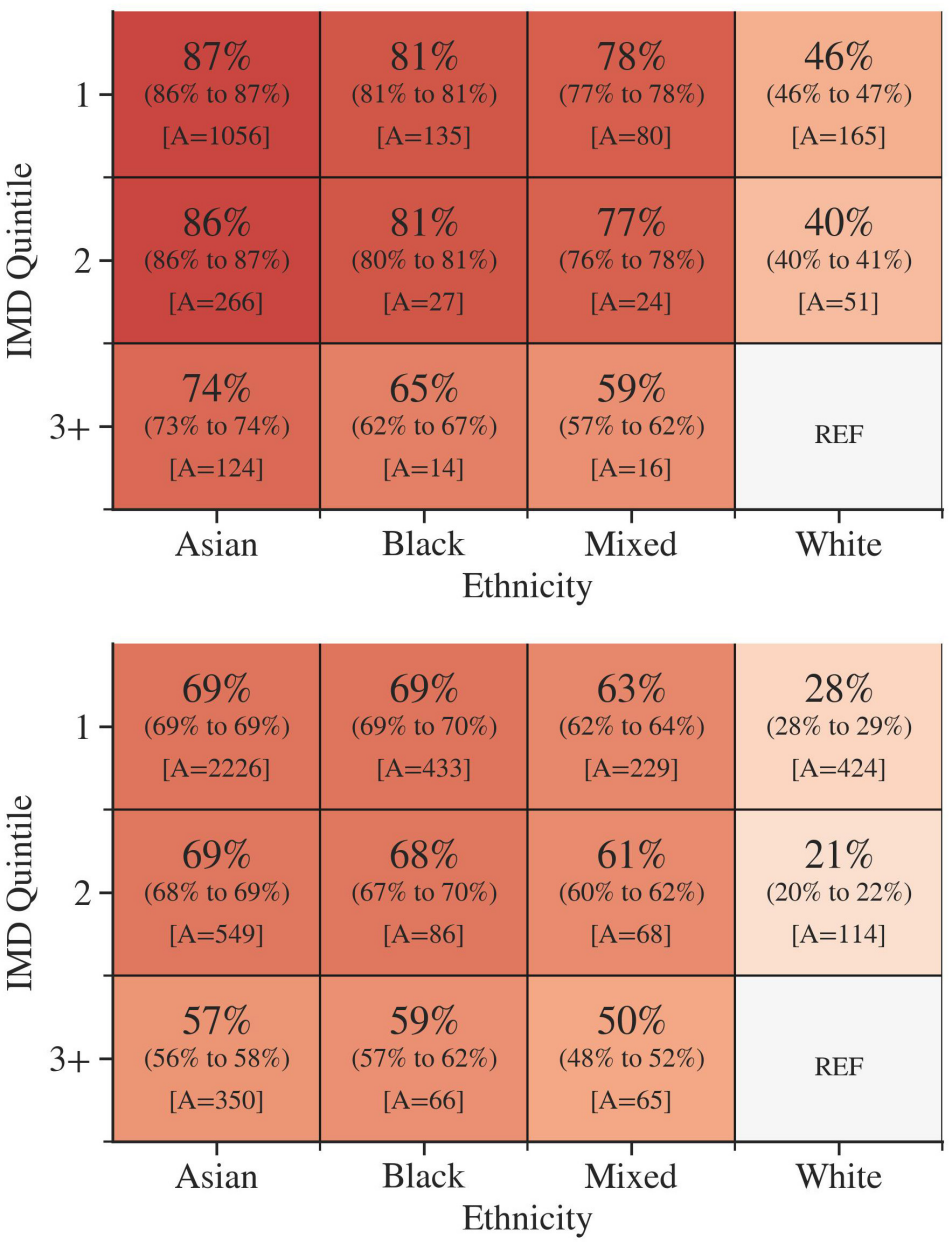
The group-specific adjusted attributable fractions for diabetes **(Top)** and pre-diabetes **(Bottom**) according to socioeconomic deprivation and ethnicity. 95% confidence intervals are in parentheses. A is the number of excess outcomes in the data set that would have been avoided. Darker colors indicate a higher group attributable fraction.

The group-specific AFs for diabetes and pre-diabetes attributable to ethnicity and IMD quintile were calculated, as illustrated in Figure 1. The distribution of subjects by ethnicity and IMD quintile is available in the Supplementary Figure S4. These AFs demonstrated the proportion of diabetes or pre-diabetes cases that would have been avoided if all attendees had the same prevalence as the least deprived White attendees. The AFs were substantially higher among attendees from more deprived areas and ethnic minority groups. The AF for diabetes was highest among Asian ethnicity residing in IMD quintile 1 [87%, 95% CI = (86%, 87%)]. Black [81%, (81%, 81%)] and Mixed [78%, (77%, 78%)] ethnicities in IMD quintile 1 closely followed. Similarly, higher AFs for pre-diabetes were observed in the most deprived quintile in Asian [69%, (69%, 69%)], Black 69%, (69%, 70%) and Mixed [63%, (62%, 64%)] ethnicity.

### 3.3 Multivariate analysis

**Figure 2:**
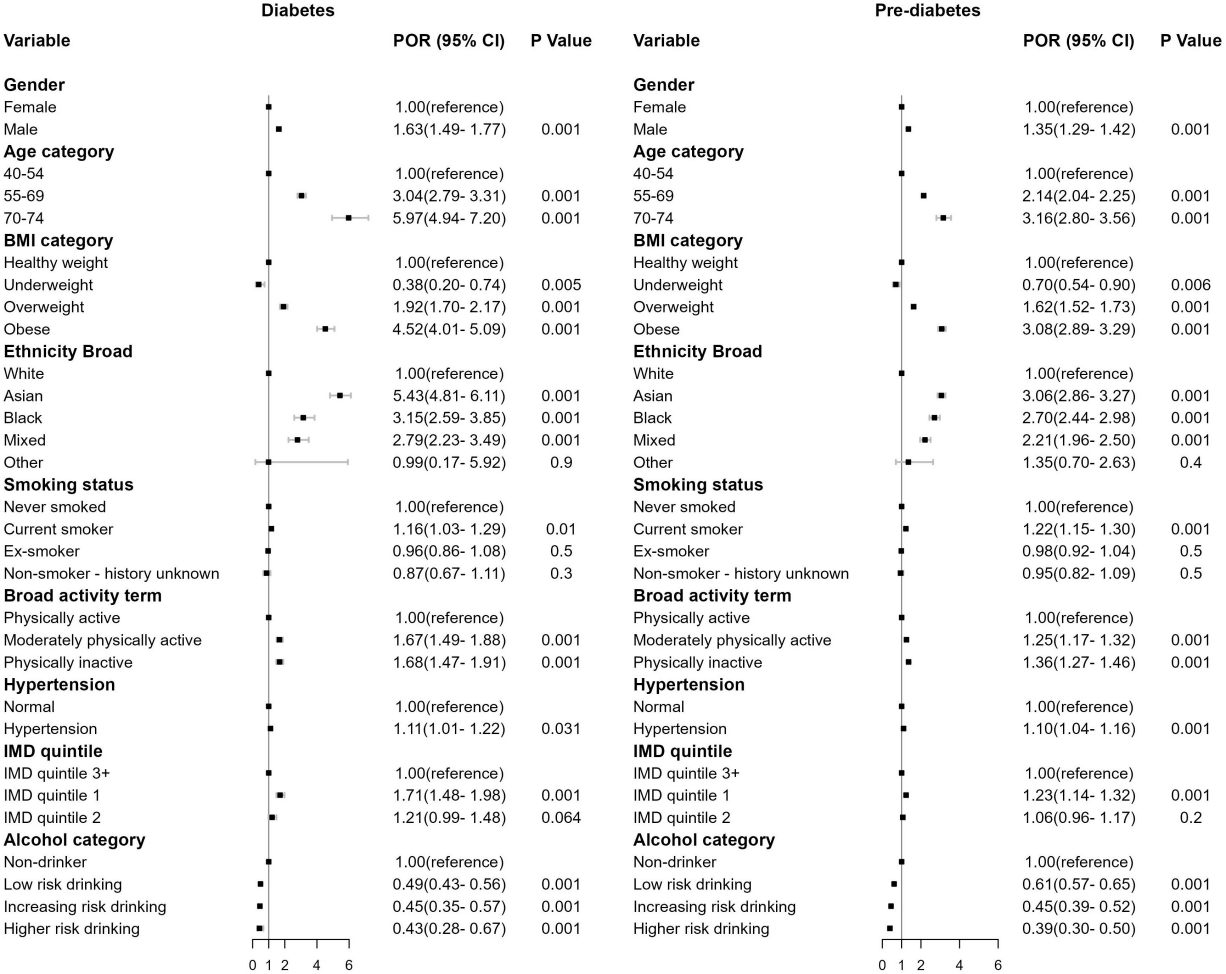
Forest plot of the regression coefficient in prevalence odds ratios (POR), 95% confidence interval (CI) and *p*-value for diabetes **(left)** and pre-diabetes **(right)** compared to the baseline non-diabetes (omitted).

An MLR model was employed to investigate the association between the factors influencing individual and their respective outcomes in terms of diabetes and pre-diabetes. Figure 2 presents the estimated POR for diabetes and pre-diabetes regarding various determinants, including ethnicity, IMD quintile and other risk factors.

Males exhibited higher risk than females [diabetes: POR = 1.63, (1.49, 1.77), *p* < 0.001; pre-diabetes: POR = 1.35, (1.29, 1.42), *p* < 0.001]. Obese individuals demonstrated the strongest association compared to individuals in other BMI categories [diabetes: POR = 4.52, (4.01, 5.09), *p* < 0.001; pre-diabetes: POR = 3.08, (2.89, 3.29), *p* < 0.001]. Older individuals aged 70–74 showed increased risk [diabetes: POR = 5.97, (4.94, 7.20), *p* < 0.001; pre-diabetes: POR = 3.16, (2.80, 3.56), *p* < 0.001], as did those aged 55–69 [diabetes: POR = 3.04, (2.79, 3.31), *p* < 0.001; pre-diabetes: POR = 2.14, (2.04, 2.25), *p* < 0.001]. Current smokers [diabetes: POR = 1.16, (1.03, 1.29), *p* < 0.01; pre-diabetes: POR = 1.22, (1.15, 1.30), *p* < 0.001], physically inactive individuals [diabetes: POR = 1.68, (1.47, 1.91), *p* < 0.001; pre-diabetes: POR = 1.36, (1.27, 1.46), *p* < 0.001], and those with hypertension [diabetes: POR = 1.11, (1.01, 1.22), *p* < 0.001; pre-diabetes: POR = 1.10, (1.04, 1.16), *p* < 0.001] were also associated with increased risk.

Ethnicity was a significant predictor for both diabetes and pre-diabetes (*p* < 0.001). Asian ethnicity showed the strongest association relative to White ethnicity [diabetes: POR = 5.43, (4.81, 6.11), *p* < 0.001; pre-diabetes: POR = 3.06, (2.86, 3.29), *p* < 0.001], the effect size were then followed by Black and Mixed ethnicities.

Individuals residing in socioeconomically disadvantaged areas exhibited an elevated risk of having diabetes and pre-diabetes HbA1C level. The risk of being diabetes and pre-diabetes for individuals who lived in IMD quintile 1 areas were 1.71 [(1.48, 1.98), *p* < 0.001] and 1.23 [(1.14, 1.32), *p* < 0.001] times that of individuals who lived in the least deprived areas in IMD quintile 3+.

Alcohol consumption demonstrated a protective effect against diabetes and pre-diabetes HbA1C level. In comparison to non-drinkers, individuals who engaged in low-risk drinking, increasing-risk drinking, and higher-risk drinking exhibited a reduced likelihood of having diabetes [POR = 0.49, (0.43, 0.56), *p* < 0.001; POR = 0.45, (0.35, 0.57), *p* < 0.001; POR = 0.43, (0.28, 0.67), *p* < 0.001] and pre-diabetes HbA1C level [POR = 0.61, (0.57, 0.65), *p* < 0.001; POR = 0.45, (0.35, 0.57), *p* < 0.001; POR = 0.39, (0.30, 0.50), *p* < 0.001]. No multicollinearities were found between alcohol and other variables (Supplementary Figure S8).

**Figure 3:**
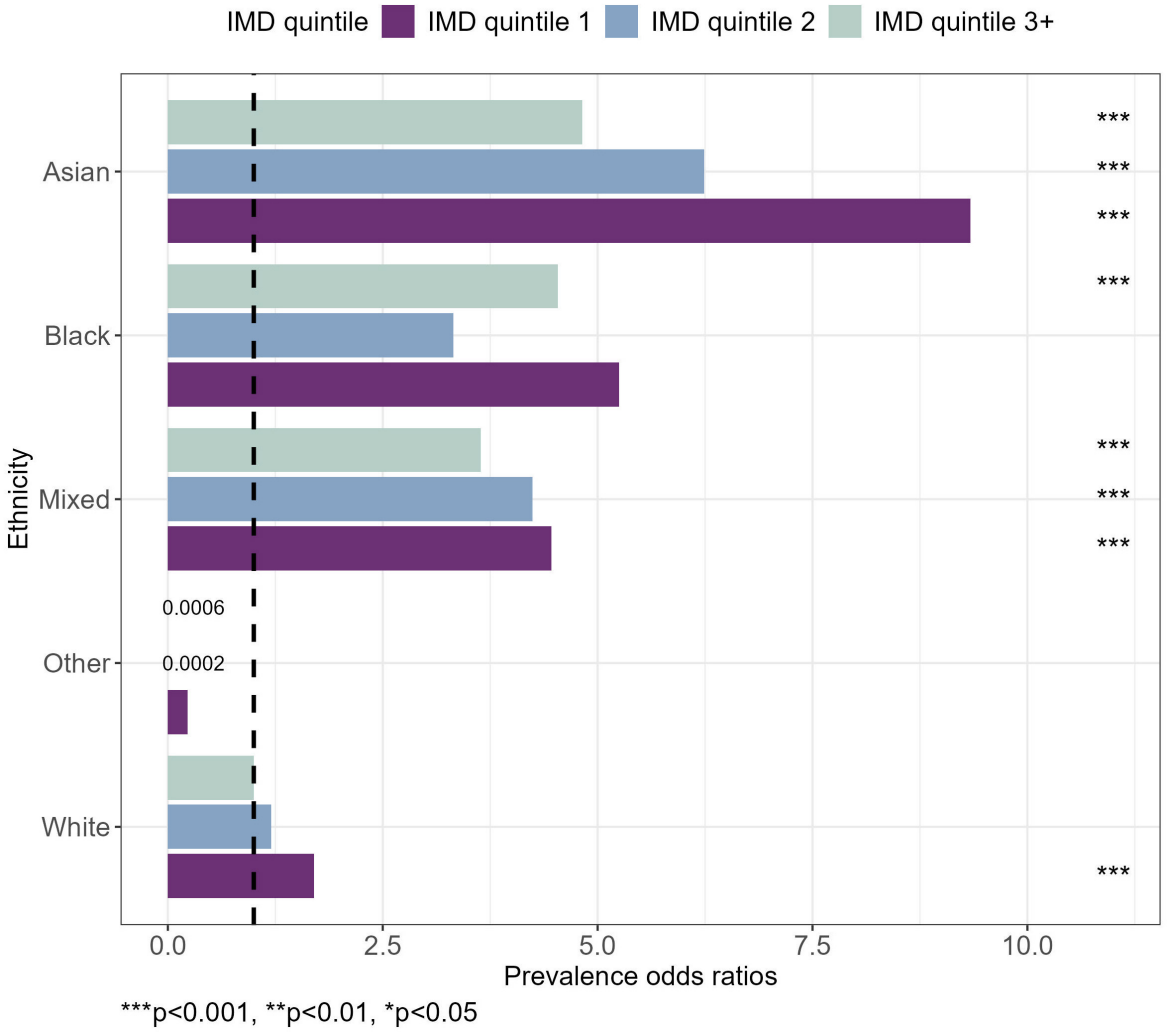
Prevalence odds ratios for diabetes, by IMD quintile and ethnicity. Taking into account gender, age, BMI category, smoking status, broad activity term, hypertension and alcohol category. ***p < 0.001, **p < 0.01, *p < 0.05.

Figure 3 presents the net effects of interaction between the IMD quintile and ethnicity on diabetes. A table containing estimates and corresponding standard errors has been included in the Supplementary Table S1. For pre-diabetes, most interaction effects were found to be statistically non-significant as presented in the Supplementary Table S2.

The combination of having an Asian ethnicity and living in IMD quintile 1 areas had the strongest association with a POR of 9.34 [(4.30, 19.84), *p* < 0.001] relative to White ethnicity living in the least deprived areas. The effect sizes for Asian ethnicity were followed by Asian ethnicity living in IMD quintile 2 and IMD quintile 3+ areas. A similar pattern was observed for Mixed Ethnicity, whereby the POR reduced for IMD quintile 2 and then again for IMD quintile 3+ (Figure 3).

The coefficients for “Other” ethnicity could potentially be biased or collinear because of a limited number of cases when combining categories into smaller subgroups, such that the POR was inflated as shown in the Supplementary Table S3.

## 4 Discussion

To date, the social determinants of health have not been fully examined with respect to the onset and development of diabetes. The present study adds new knowledge to the literature base regarding the risk of having diabetes and pre-diabetes among a representative cohort of Birmingham's HCs. The key findings are that multiple socioeconomic and demographic factors contribute to the risk of elevated HbA1c levels among middle-aged and older adults. Asian patients were at the greatest risk of diabetes and pre-diabetes, followed by Black and Mixed ethnic groups. Findings also showed that socioeconomic deprivation increased the risk of diabetes and pre-diabetes. Interaction effects between ethnicity and deprivation were also found for diabetes with Asian attendees living in the most deprived areas exhibiting the greatest risk. An unexpected finding was that all levels of self-reported alcohol consumption were a protective factor for diabetes and pre-diabetes compared to non-drinkers.

### 4.1 Ethnicity

The present study identified a higher risk of diabetes and pre-diabetes among individuals of Asian and Black ethnicities, consistent with the extant literature (48–57). Two UK studies reported a higher prevalence of diabetes among individuals of Asian, Black, and Mixed ethnicities, with Asian groups showing a significantly greater likelihood of being diagnosed with diabetes (48, 49). Another UK-based study observed that individuals of South Asian or Mixed-Race ethnicity have an increased risk of progression from pre-diabetes to Type 2 diabetes (50). Asian and particularly South Asian people are found to have higher levels of insulin resistance compared to White Europeans which might be attributed to the central adiposity-linked insulin resistance in South Asians (51, 52). After controlling for BMI, South Asians tend to have significantly higher levels of body fat concentrated in the upper body and abdominal region compared to White Europeans (53). Another potential explanation could be rooted in the genetic predisposition of Asians, where metabolically thrifty genes may have provided survival advantages during periods of subsequent famine (54, 55). The elevated risk observed in Black ethnicity can be attributed to similar factors. Findings from studies indicate that Black individuals tend to exhibit lower rates of insulin clearance in comparison to White ethnicity (56, 57).

### 4.2 Deprivation

Deprivation is closely linked to factors such as obesity and physical inactivity, which significantly contribute to diabetes risk and its complications (58). Furthermore, individuals who live in the most deprived areas in the UK are 2.5 times more likely to have diabetes at any age (59) and that also highlighted socioeconomic deprivation raises the risk of progression from pre-diabetes to diabetes (50). These findings align with the results of the present study, where socioeconomic deprivation increased the risk of diabetes and pre-diabetes. The relationship between deprivation and health is multifaceted. Plausible mechanisms explaining the link between deprivation and raised HbA1c levels include limited income, lower health literacy, housing instability, and reduced food access (60). These socioeconomic factors negatively influence an individual's ability to effectively manage their health. In areas of greater deprivation, individuals often contend with reduced access to nutritious food options (60). This is further exacerbated by the fact that the density of fast food outlets in England's poorest areas is five times higher compared to the most affluent areas (61). Studies have reported that the availability of fast food establishments is linked to a higher prevalence of diabetes (62, 63). Birmingham has the second highest count of fast food outlets among all local authority areas at a density of 77.7 per 100,000 population (61). The higher count of these outlets imposes limited nutritious options and may encourage unhealthy eating habits, thereby demonstrating the pervasive nature of deprivation in raising the risk of elevated HbA1c levels. In addition, socioeconomic deprivation also significantly influences the development of complications associated with Type 2 diabetes. A recent study found that social deprivation is a risk factor for the development of Type 2 diabetes related foot diseases, with significantly higher risks in the most deprived quintile compared to the least deprived quintile (64). This highlights socioeconomic deprivation not only increases the risk of developing Type 2 diabetes but also elevates the risk of its severe complications. The potential costs associated with these severe complications could have been significantly reduced if preventive strategies were implemented to identify at-risk individuals in deprived communities.

Since the interaction between ethnic disparities and socioeconomic deprivation is complex, it is difficult to disentangle these two factors (27). The influence of socioeconomic deprivation which is closely associated with lack of resources and opportunities, plays a pivotal role in elucidating the patterns of ethnic disparities and health status. The English indices of deprivation 2019 (65) reported that among all ethnic groups, individuals of Asian ethnicity (15.7%) were most likely to live in the most in the most deprived 10% of neighborhoods, followed closely by individuals of Black ethnicity (15.2%). In the present study, among all ethnic groups, Black and Asian attendees were found to live in the most deprived quintile in 64 and 65% of cases, respectively, compared to 23% for White attendees (Supplementary Figure S4). The interactions of these factors were in line with the results of a recent and similar study by Nagar et al. (28). However, in distinction to prior research, the present study is less prone to bias due to the control of confounding factors such as BMI, ensuring that the observed trends across ethnicities reflect true associations rather than being influenced by other clinical or lifestyle factors. In addition, the attributable fraction analysis in the current study suggested that 1,056 cases of diabetes among Asian ethnicity residing in the most deprived areas would have been avoided if they had the same prevalence as White ethnicity in IMD quintile 3+ areas. Based on a 2017 study of the costs of diabetes treatment pathways, avoiding these cases would save the NHS ~£65,000 per year from medication costs alone (66). The present findings therefore indicate that there is an interaction effect between ethnicity and socioeconomic deprivation with regards to the development of diabetes, with Asian ethnicity and living in the most deprived areas predisposing individuals to a disproportionately greater risk.

### 4.3 Alcohol consumption

The unexpected protective effect of alcohol consumption remains unclear and inconclusive. Previous meta-analyses (67–69) and systematic review (70) indicated consistent findings of U-shaped association, meaning that light and moderate alcohol consumption were associated with reduced diabetes risk but heavy drinking increased the risk of diabetes. This can be explained by a mechanism related to the triglyceride metabolism, wherein low and moderate alcohol consumption enhances insulin sensitivity, while heavy drinking exacerbates liver steatosis and increases triglyceride levels, thereby counteracting potential benefits (71–73). Furthermore, heavy drinking displays a stronger correlation with body fat gain, particularly in adolescents and older adults (74). The present study is in contrast to previous research, as all levels of drinking were associated with reduced risk of diabetes and pre-diabetes. When controlling for BMI, a risk reduction in diabetes and pre-diabetes was still observed. This might raise the concern that obesity could be part of the causal pathway between alcohol consumption and diabetes. However, short-term alcohol consumption may increase insulin sensitivity via leptin and adiponectin from adipose tissue without affecting body weight gain and body fat mass (75). Longer-term alcohol consumption may lead to different outcomes. A literature review suggested that chronic alcohol consumption may induce leptin resistance due to prolonged elevation of leptin, potentially contributing to the development of diabetes (76). The same literature review reported a rat model of diabetes and observed that chronic heavy alcohol consumption reduced Brain-derived neurotrophic factor (BDNF) levels. The reduction in BDNF-impaired hippocampal long-term potentiation (LTP) is responsible for cognitive functions and insulin sensitivity, which may lead to diabetes. Despite these reductions in BDNF levels, diabetic rats with chronic heavy alcohol consumption were found to have less weight gain than diabetic rats that never consume alcohol. These findings suggest that the pathophysiological pathway of alcohol consumption that leads to diabetes may be independent of body weight gain. The current study found no collinearity between alcohol consumption and BMI category (Supplementary Figure S8) and thus, BMI was unlikely to be part of the causal pathway. Additionally, alcohol consumption was observed to be higher among HC attendees of White ethnicity residing in less deprived areas (Supplementary Figure S6). While there appears to be a correlation in such instances, no statistically significant relationship was found (Supplementary Figure S7). This disparate finding could be attributed to utilizing non-drinkers as the reference category, as this group is non-homogeneous and includes former drinkers who might have poorer health, thereby overestimating the reduction in risk of different levels of alcohol use (77, 78). Another explanation could be using HbA1c as the sole biomarker to classify non-diabetes, diabetes and pre-diabetes subjects. A systematic review and meta-analysis (79) and recent study (80) confirmed that moderate drinking and heavy drinking were associated with reduced HbA1c levels. Reduction in hemoglobin content and concentration in red blood cell cytoplasm from ethanol exposure may partially explain the overall effect of alcohol on HbA1c (81). Therefore, classification error could have emerged in the current study when adopting the universal cut-off of HbA1c levels. In addition, individuals with pre-existing health conditions such as chronic kidney disease (CKD) are not eligible for a health check. Since alcohol consumption is a risk factor for many of these conditions, it is possible that those who are eligible for a HC are biased toward those whose health is less affected by alcohol consumption. If they were included in this study, the results could be altered and the protective effect of alcohol might not be observed.

### 4.4 Policy implications

This study has important public health implications for Birmingham. The amplified risk of diabetes and pre-diabetes among individuals who are specifically Asian ethnicity and those residing in the most deprived areas suggests that incorporating ethnicity and socioeconomic deprivation into diabetes risk stratification could be highly beneficial. Targeted public health services that focus on these key factors may effectively reduce health inequalities in Birmingham.

### 4.5 Strengths and limitations

Strengths of this study include a sophisticated and comprehensive analytical approach enabled the complex interaction between ethnicity and deprivation to be examined, strengthening the evidence base on socioeconomic and demographic factors that contribute to the development of diabetes. This study provides a representative picture of a substantial sample of adults aged 40–74, enabling the comparison of the likelihood of being diabetic and pre-diabetic concerning demographic characteristics, behavioral risk factors, ethnicity, and deprivation. In addition, this study investigates this topic within the middle-aged and older adults of Birmingham, aiming to improve the analysis and bridge the existing research gap for these age groups, especially in the context of Birmingham, known for its high levels of deprivation and cultural and ethnic diversity.

The present study also had limitations that should be acknowledged and the findings should be interpreted with caution. Firstly, the recruitment design of NHS HC only includes individuals aged 40–74 years without pre-existing health conditions, and that may under-represent the true prevalence of diabetes. This study only includes data on individuals who accessed NHS HC and does not capture those who were eligible but declined or did not participate, limiting the ability to investigate the accessibility across different socioeconomic and ethnic groups. Furthermore, HCs uptake varies across different ethnic groups, this limits the programme's ability to fully capture the health status of the broader population. Secondly, the data set spans the period during the COVID-19 pandemic, which may have influenced the demographic profile of the HC attendees. Thirdly, missing ethnicity records posed a significant challenge and may have biased estimates. While multiple imputation was employed to compensate for the potential bias, it does not ensure absolute accuracy. Moreover, since the data were anonymised, adjustments for household clustering could not be performed, potentially resulting in narrower confidence intervals than would otherwise be obtained. In addition, this study adopted a cross-sectional design, thus only examining the associations between determinants and the disease rather than facilitating causal inference of these determinants. The diagnosis of diabetes and pre-diabetes was based solely on the hemoglobin A1C test without the use of an oral glucose tolerance test (OGTT) and fasting plasma glucose (FPG) test. These gold standard tests are more sensitive and accurate in detecting pre-diabetes and diabetes (82, 83). Therefore, the reliance on HbA1c levels alone may lead to misclassification of diabetes or pre-diabetes cases. Moreover, IMD scores were not available for each patient's address, rather this study used the IMD associated with the patient's GP postcode as a proxy variable. Furthermore, comparing patients to those in IMD quintile 3+ categories likely reduced the observed impact of deprivation, which may underestimate the true impact of socioeconomic deprivation on diabetes outcomes. The study also did not use the NICE obesity definition, which led to heterogeneity within the obesity group. Concerns may arise about higher BMI due to muscle hypertrophy as a result of a better diet and increased engagement in physical activity, especially among individuals who live in more affluent areas. However, as the sample comprised middle aged and older adults from 40 to 74 years, it is unlikely that BMI is influenced by muscle mass. This is because muscle mass declines after the age of 30 at a rate between 3 and 8% per decade and the rate is even higher after the age of 60 (84, 85). Furthermore, muscle loss in older individuals is often accompanied by intramuscular fat accumulation, with the proportion of intramuscular fat relative to the total muscle cross-sectional area being ~2.5 times higher in older adults compared to younger individuals (86). Lastly, self-reported smoking status, physical activity, and alcohol consumption may introduce social-desirability bias (87).

## 5 Conclusions

The findings of this study highlight a significant association between ethnicity, socioeconomic deprivation, and various determinants with an increased risk of diabetes and pre-diabetes. Notably, the interaction between ethnicity and socioeconomic deprivation magnifies these effects. These findings underline the relevance of ethnic health disparities within the context of Birmingham, thereby emphasizing the need for target interventions and policies aimed at mitigating these inequalities. The protective effect of alcohol consumption remains unclear. This study does not intend to advocate alcohol consumption as a preventive measure against diabetes, as it is well-established that alcohol consumption is linked to the development of various cardiovascular and other chronic diseases. Further work is needed to unpack the subcomponents of socio-economic deprivation at a more granular level, including factors such as education, access to green space, and employment status, to better understand their correlations with diabetic risk.

## Data Availability

The data analyzed in this study is subject to the following licenses/restrictions. Individuals can be identified from the raw data and therefore the data can not be made publicly available in compliance with GDPR. Requests to access these datasets should be directed to Midland and Lancashire Commissioning Support Unit.
