# Supplementary material for: Socioeconomic and ethnic inequalities increase the risk of type 2 diabetes: an analysis of NHS health check attendees in Birmingham
Source: Front Public Health. 2024 Nov 27;12:1477418. doi: 10.3389/fpubh.2024.1477418 (PMC11631903; doi:10.3389/fpubh.2024.1477418)
Supplement: Supplementary file 1 [file Data_Sheet_1.pdf]

Supplementary Material

1 UPTAKE OF HC PROGRAMME

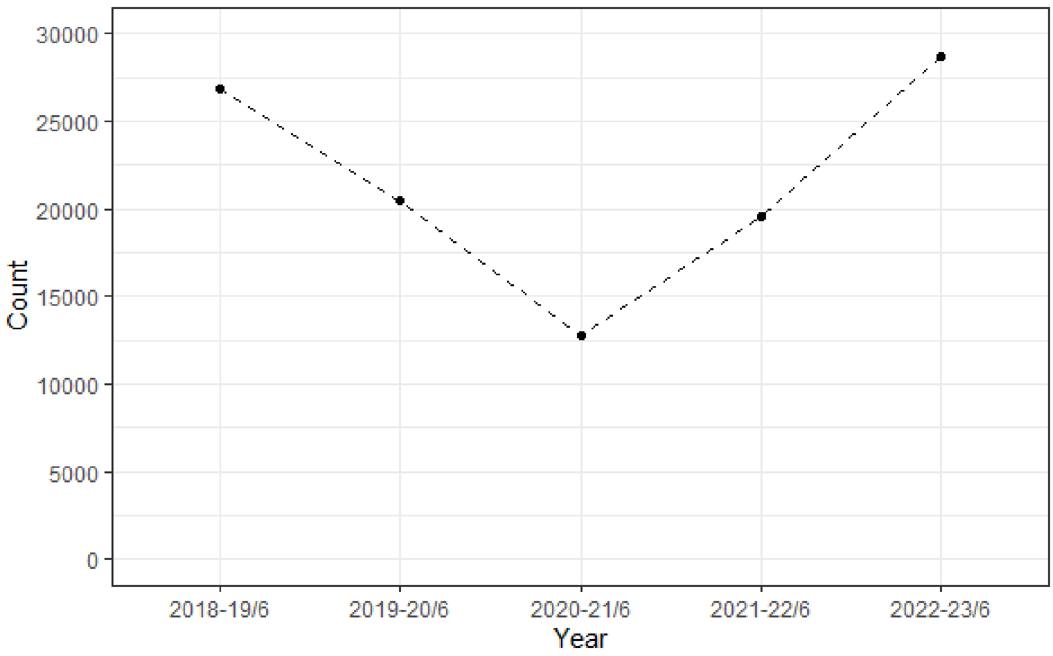

Figure S1. Uptake of HC prgramme from 2018/6 to 2023/6

The COVID-19 pandemic influenced the uptake of NHS HC programme as shown in the graph above. The implementation of national lockdowns restricted all ”non-essential” businesses and activities, including the delivery of HCs. Consequently, a substantial decline in the uptake of HCs was observed. The reduced uptake of HCs may not accurately reflect the demographic profile of the overall population. Further, the delayed delivery of HCs may have implications for the health conditions of the eligible individuals, potentially affecting factors such as elevated HbA1c levels. This concern was raised in the limitation, as the impact of pandemic may have exacerbated the health conditions of the eligible population.

2 OTHER INDEPENDENT VARIABLES

Smoking status was recorded as the number of cigarettes consumed each day with 96 unique status within the data set. To effectively analyse this variable, the smoking status was categorised into ‘Current smoker’, ‘Never smoked’, ‘Non-smoker’ and ‘Ex-smoker’. ‘Never smoked’ is the reference category.

Physical activity. The General Practice Physical Activity Questionnaire (GPPAQ) was used as a validated tool to measure the physical activity index. The output was given as active, moderately active, moderately inactive and inactive. This study categorised the output into 2 variables, those who reported active and moderately active were coded as ‘active’. Those who reported moderately inactive and inactive were coded as ‘inactive’. ‘active’ is the reference category.

Alcohol category. The number of units of alcohol consumed per week was included as a discrete variable in the data set. It recorded the amount of alcohol consumption per week by the HC attendees. The measurement of alcohol units aligns with the definition from the NHS where one unit equals 10ml of alcohol NHS (2023). This was then categorised into ‘Non-drinkers’, ‘Low-risk drinking’ if alcohol units per week below and equal to 14, ‘Increasing risk drinking’ if alcohol units per week between 15 and 35 and ‘High-risk drinking’ if alcohol units per week above 35 Department of Health and Social Care (2021). ‘Non-drinkers’ is the reference category.

Gender was recorded as a binary variable with two categories: ‘Male’ and ‘Female’. It is important to note that non-binary or other gender identities were not provided in the data set. ‘Female’ is the reference category.

Age category. Age was included as a discrete variable. The data exclusively comprised HC attendees within the age range of 40 to 74 years.

BMI category. The body mass index (BMI) was recorded as a continuous variable and calculated from height and weight. HC attendees with BMI below 18.5 were categorised into ‘Underweight’, BMI between 18.5 and 24.9 were categorised into ‘Healthy weight’, BMI between 25 and 29.9 were categorised into ‘Overweight’ and BMI above and equal to 30 were categorised into ‘Obese’.

Hypertension was defined based on systolic blood pressure (SBP) and diastolic blood pressure (DBP). HC attendees were categorised into hypertension when the SBP is ≥ 140 mm Hg and/or their diastolic blood pressure (DBP) is ≥ 90 mm Hg.

3 RISK FACTOR PREVALENCE

The prevalence of the different risk factors studied here are given in Figures. S2. All risk factor prevalences for each ethnicity-IMD combination are compared to the prevalence for White patients in the least deprived areas (IMD Quintile 3+) using a two-sample t-test. The significance of absolute differences are indicated using standard asterisk notation [1].

Smoking status (Figure. S2A) was recorded for 99.9% of HC attendees. Of these, $18.5 \pm 0.5\%$ were recorded as current smokers. Those living in the most deprived quintile were predictably much more likely to be current smokers ($20.3 \pm 0.3\%$) than those in the three least deprived quintiles ($13.0 \pm 0.4\%$). Smoking prevalence was highest amongst those with an Other ($25.3 \pm 7.6\%$), Mixed ($23.3 \pm 1.4\%$), or White ($22.2 \pm 0.4\%$) ethnicity.

Height and weight data was available to calculate BMI category (Figure. S2B) for 99.5% of HC attendees. Of these, $69.6 \pm 0.3\%$ were overweight or obese. Those living in the most deprived quintile had a much higher prevalence ($71.7 \pm 0.3\%$) than those in the three least deprived quintiles ($64.5 \pm 0.6\%$). Attendees with a Black ethnicity ($76.0 \pm 1.4\%$) had the highest prevalence and patients with an Asian ethnicity ($66.6 \pm 0.7\%$) had the lowest.

Alcohol consumption (Figure. S2C) was recorded for 76.5% of HC attendees. Of these, $9.1 \pm 0.6\%$ were reported as having either increasing or higher risk levels of alcohol consumption. Those living in the in the three least deprived quintiles ($13.7 \pm 0.5\%$) or second most deprived quintile ($14.0 \pm 0.5\%$) reported significantly higher levels of alcohol consumption than those living in the most deprived quintile ($6.5 \pm 0.2\%$). The prevalence of increasing or higher risk levels of alcohol consumption where highest amongst those with a White ethnicity ($15.0 \pm 0.4\%$).

Physical activity levels (Figure. S2D) were recorded for 89.1% of HC attendees. Of these, $19.4 \pm 0.5\%$ were reported as being physically inactive. The prevalence of physical inactivity was highest amongst those living in the most deprived quintile ($21.8 \pm 0.3\%$) and lowest amongst those living in the second most deprived quintile ($12.2 \pm 0.5\%$). Additionally, the prevalence of physical inactivity was highest amongst those with an Asian ethnicity ($23.6 \pm 0.6\%$) and lowest amongst those with a White ethnicity ($16.3 \pm 0.4\%$).

Finally, hypertension status (Figure. S2E) was recorded for every HC attendee. Patients were recorded as having hypertension $20.4 \pm 0.5\%$ of cases. The prevalence of hypertension was highest amongst those living in the second most deprived quintile ($22.5 \pm 0.6\%$) and lowest amongst those living in the most deprived quintile ($19.9 \pm 0.3\%$). Those with a White ($22.9 \pm 0.4\%$), Black ($22.7 \pm 1.3\%$), Mixed ($21.9 \pm 1.4\%$), or Unknown ($20.3 \pm 0.3\%$) ethnicity had a significantly higher prevalence of hypertension than those with an Asian ($15.2 \pm 0.5\%$) or Other ($12.6 \pm 5.9\%$) ethnicity.

[1] 1, 2 and 3 *'s refer to $p < 0.1$, $p < 0.05$, and $p < 0.01$ respectively.

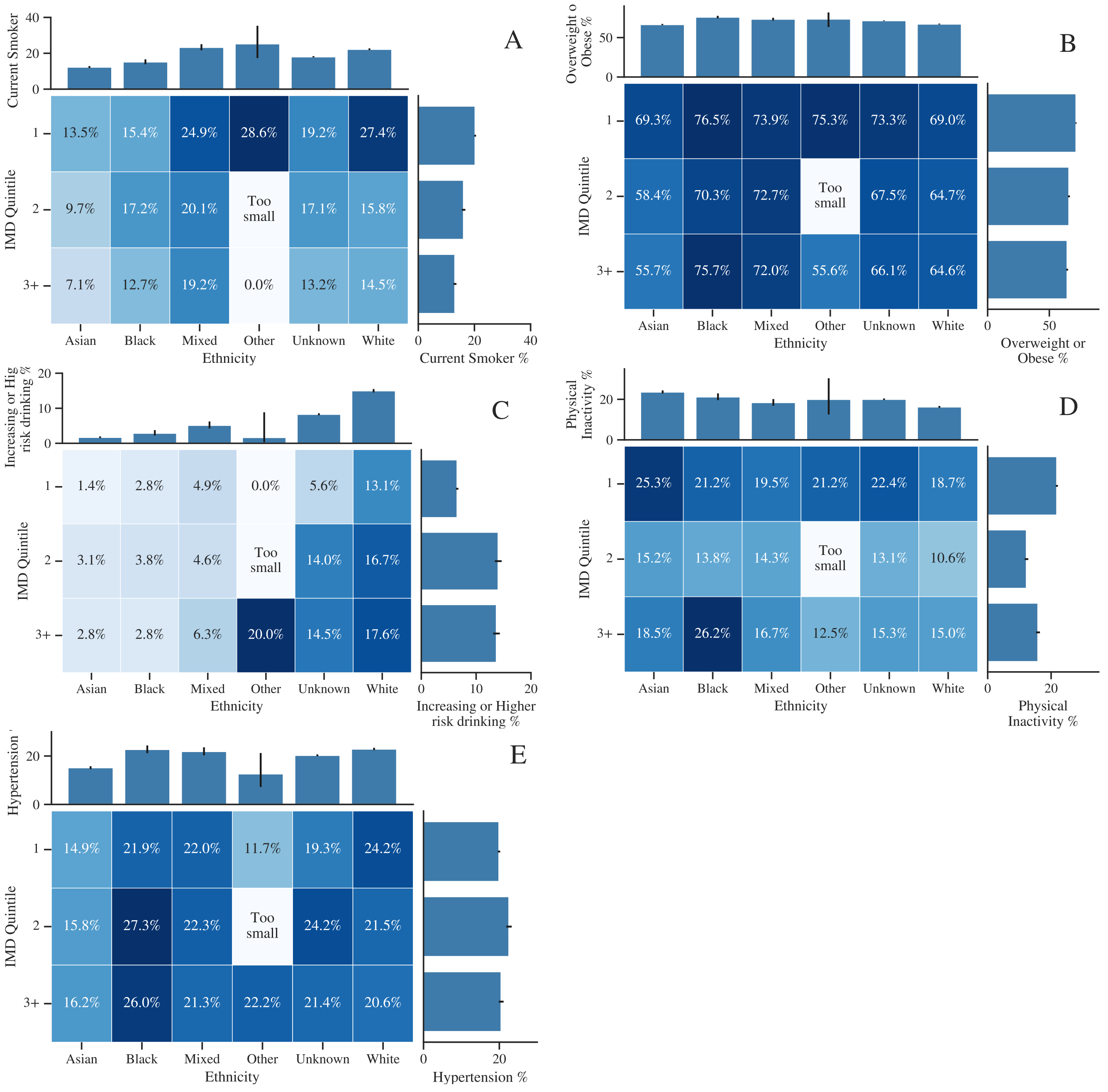

Figure S2. Risk factor prevalence for each IMD and broad ethnicity combination. A: Current smoker, B: overweight or obese, C: increasing or higher risk drinking, D: physical inactivity, E: hypertension. Error bars indicate the 95% confidence interval calculated using the Wilson Score method. Numbers less than five have been supressed.

4 INTERACTION EFFECTS

In probability theory, the Bienayme's identity states that the variance of the sum of j random variables equal to the sum of all Variances and 2 times the covariances. Assuming $X = X_1 + X_2 + X_3 + \ldots + X_j$ for any $i \neq j$ and X_i, X_j are independent :

$$Var(X) = \mathbb{E}(X_1 + ... + X_j)^2 - (\bar{X}_1 + ... + \bar{X}_j)^2 \tag{S1}$$

The above can be expanded to:

$$\begin{aligned} Var(X) = \sum_{i=1}^{n} \mathbb{E}[X_i^2] - \sum_{i=1}^{n} \bar{X}_i^2 + 2 \sum_{1 \leq i < j \leq n} \mathbb{E}[X_i X_j] \\ - 2 \sum_{1 \leq i < j \leq n} \bar{X}_i \bar{X}_j \end{aligned} \tag{S2}$$

Following equation S2, if X_i, X_j are not independent:

$$Var(X) = \sum_{i=1}^{n} Var(X_i) + 2 \sum_{1 \leq i < j \leq n} Cov(X_i, X_j) \tag{S3}$$

This study explored the net effect of two variables, comprising both the the main effects and the interaction effects, resulting in a total of three coefficients within the model for interpretation. Given that equation S3 is the variance of sum of n random variables, when n=3 ($X = X_1 + X_2 + X_3$), the variance of the sum is:

$$\begin{aligned} Var(X) &= \mathbb{E}(X_1 + X_2 + X_3)^2 - (\bar{X}_1 + \bar{X}_2 + \bar{X}_3)^2 \\ &= \mathbb{E}[X_1^2] - \bar{X}_1^2 + \mathbb{E}[X_2^2] - \bar{X}_2^2 + \mathbb{E}[X_3^2] - \bar{X}_3^2 + \\ &2\mathbb{E}[X_1 X_2] - 2\bar{X}_1\bar{X}_2 + 2\mathbb{E}[X_1 X_3] - 2\bar{X}_1\bar{X}_3 + \\ &2\mathbb{E}[X_2 X_3] - 2\bar{X}_2\bar{X}_3 \\ &= Var(X_1) + Var(X_2) + Var(X_3) + \\ &2[Cov(X_1, X_2) + Cov(X_1, X_3) + Cov(X_2, X_3)] \end{aligned} \tag{S4}$$

Therefore, the standard error of the net effect of interaction terms is the square root of the variance of the sum in equation S4.

Table. S1 and S2 calculate the net effect of the interaction between deprivation and ethnicity. For instance, the net effect of interactions among categorical independent variables can be calculated by multiplying the *POR* of diabetic or pre-diabetic levels of HbA1c for each of the main effects and the interaction term. For instance, the *POR* of diabetic level of HbA1c for Asian who lived in IMD quintile 1 areas compared to the reference category = ($POR_{Asian} \times POR_{IMD-quintile-1} \times POR_{Asian \times IMD-quintile-1}$) = $4.82 \times 1.7 \times 1.14 = 9.34$. For groups situated within the reference category for one of the variables in the interaction but not both, the calculation is simple and straghtfoward. For example, the *POR* of diabetic

Table S1. Net effects of interaction between IMD quintile and Ethnicity in diabetic HbA1c level

	IMD Quintile 1	IMD Quintile 2	IMD Quintile 3
**Asian**	9.34***	6.24***	4.82***
	(0.39)	(0.53)	(0.19)
**Black**	5.25	3.32	4.54***
	(1.15)	(1.03)	(0.35)
**Mixed**	4.46 ***	4.24***	3.64***
	(0.26)	(0.33)	(0.32)
**Other**	0.23	0.0002	0.0006
	(1.60)	(6.23)	(2.97)
**White**	1.7***	1.2	Ref.
	(0.13)	(0.16)	

Standard errors are in parentheses
***p<0.001, **p<0.01, *p<0.05

Table S2. Net effects of interaction between IMD quintile and Ethnicity in pre-diabetic HbA1c level

	IMD Quintile 1	IMD Quintile 2	IMD Quintile 3
**Asian**	3.69	3.08	2.70***
	(1.60)	(1.55)	(0.08)
**Black**	3.09	3.36	3.66***
	(2.11)	(2.21)	(0.15)
**Mixed**	2.65	2.31	2.04***
	(2.39)	(3.77)	(0.16)
**Other**	1.59	0.004	1.63
	(1.90)	(3.35)	(1.06)
**White**	1.19**	1.02	Ref.
	(0.06)	(0.07)	

Standard errors are in parentheses
***p<0.001, **p<0.01, *p<0.05

level of HbA1c for Asian who lived in IMD quintile 3+ areas compared to the reference category = 4.82. The main effect and interaction effect of these calculations can be found in appendix table S3.

Table S3. Regression results with interaction terms

Variable	Diabetic POR[1,2]	SE[2]	Pre-diabetic POR[1,2]	SE[2]
Gender				
Female	Ref.	—	Ref.	—
Male	1.63***	0.044	1.35***	0.024
Age category				
40-54	Ref.	—	Ref.	—
55-69	3.04***	0.044	2.14***	0.025
70-74	5.98***	0.096	3.16***	0.061
BMI category				
Healthy weight	Ref.	—	Ref.	—
Underweight	0.38**	0.341	0.70**	0.132
Overweight	1.91***	0.063	1.62***	0.033
Obese	4.51***	0.061	3.08***	0.033
Ethnicity Broad				
White	Ref.	—	Ref.	—
Asian	4.82***	0.192	2.70***	0.087
Black	4.54***	0.352	3.66***	0.148
Mixed	3.64***	0.312	2.04***	0.162
Other	0.00**	2.97	1.63	1.06
Smoking status				
Never smoked	Ref.	—	Ref.	—
Current smoker	1.16**	0.056	1.22***	0.031
Ex-smoker	0.96	0.059	0.98	0.033
Non-smoker	0.87	0.129	0.95	0.071
Broad activity term				
Physically active	Ref.	—	Ref.	—
Moderately active	1.67***	0.059	1.25***	0.031
Physically inactive	1.67***	0.067	1.36***	0.037
Hypertension				
Normal	Ref.	—	Ref.	—
Hypertension	1.11*	0.048	1.10***	0.028
IMD quintile				
IMD Q3+	Ref.	—	Ref.	—
IMD Q 1	1.70***	0.136	1.19***	0.059
IMD Q2	1.20	0.163	1.02	0.069
Alcohol category				
Non-drinker	Ref.	—	Ref.	—
Low risk drinking	0.49***	0.065	0.61***	0.033
Increasing risk	0.45***	0.125	0.45***	0.068
Higher risk	0.43***	0.222	0.39***	0.135
Ethnicity Broad * IMD quintile				
Asian * IMD Q1	1.14	0.199	1.15	0.095
Black * IMD Q1	0.68	0.367	0.71*	0.160
Mixed * IMD Q1	0.72	0.343	1.09	0.178
Other * IMD Q1	221*	3.07	0.82	1.10
Asian * IMD Q2	1.08	0.259	1.12	0.135
Black * IMD Q2	0.61	0.623	0.90	0.233
Mixed * IMD Q2	0.97	0.459	1.11	0.239
Other * IMD Q2	0.22	1.92	0.00*	2.74

[1] *** $p < 0.001$, ** $p < 0.01$, *$p < 0.05$

[2] POR = prevalence odds ratio, SE = Standard Error

5 MISSING TABLE AND LITTLE'S MCAR TEST

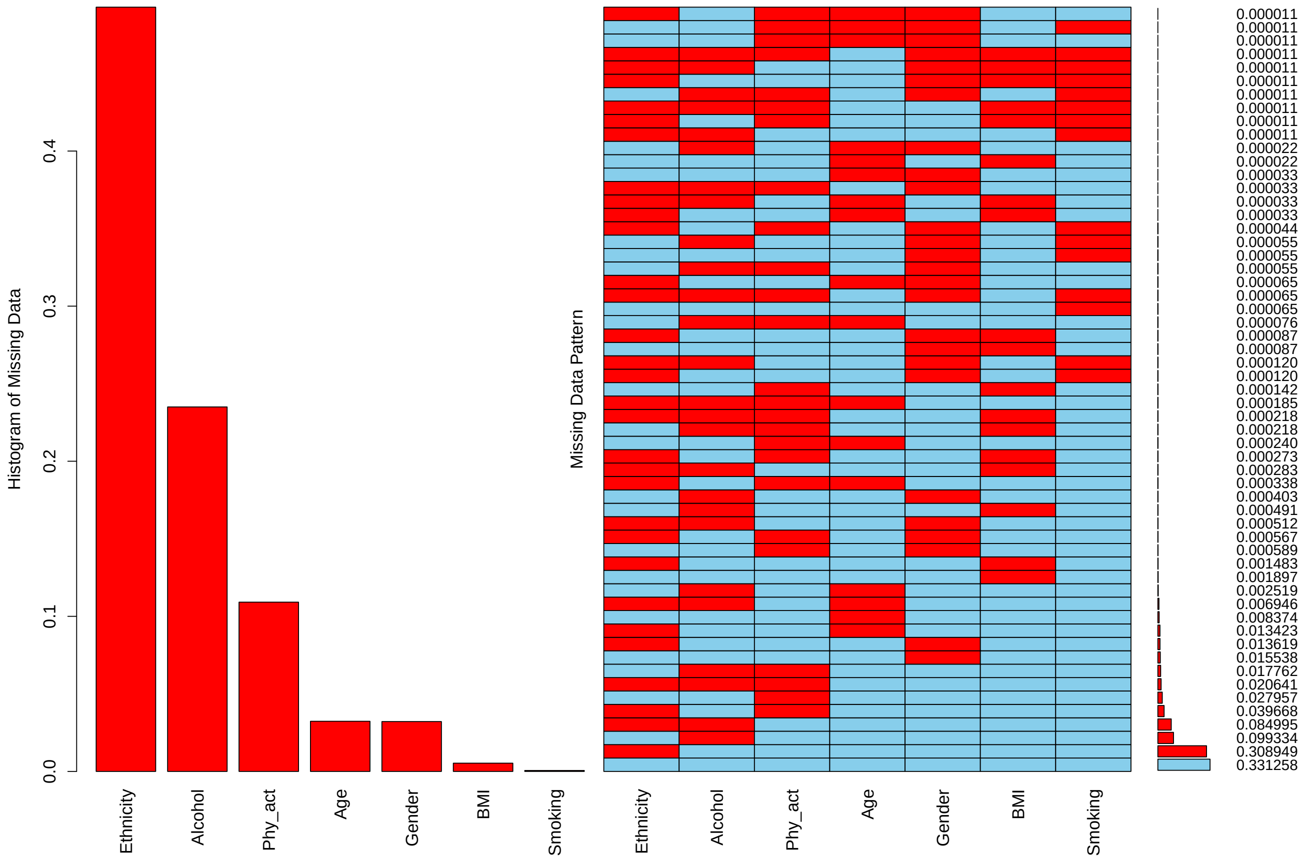

Figure S3. Histogram of the missing data and the pattern of missing values. Phy_act refers to physical activity.

Table S4. Appendix Table A1: Results of Little's MCAR Test

Statistic	Value
χ^2	10080.97
Degrees of Freedom (df)	621
p-value	< 0.001

6 ATTRIBUTABLE FRACTION

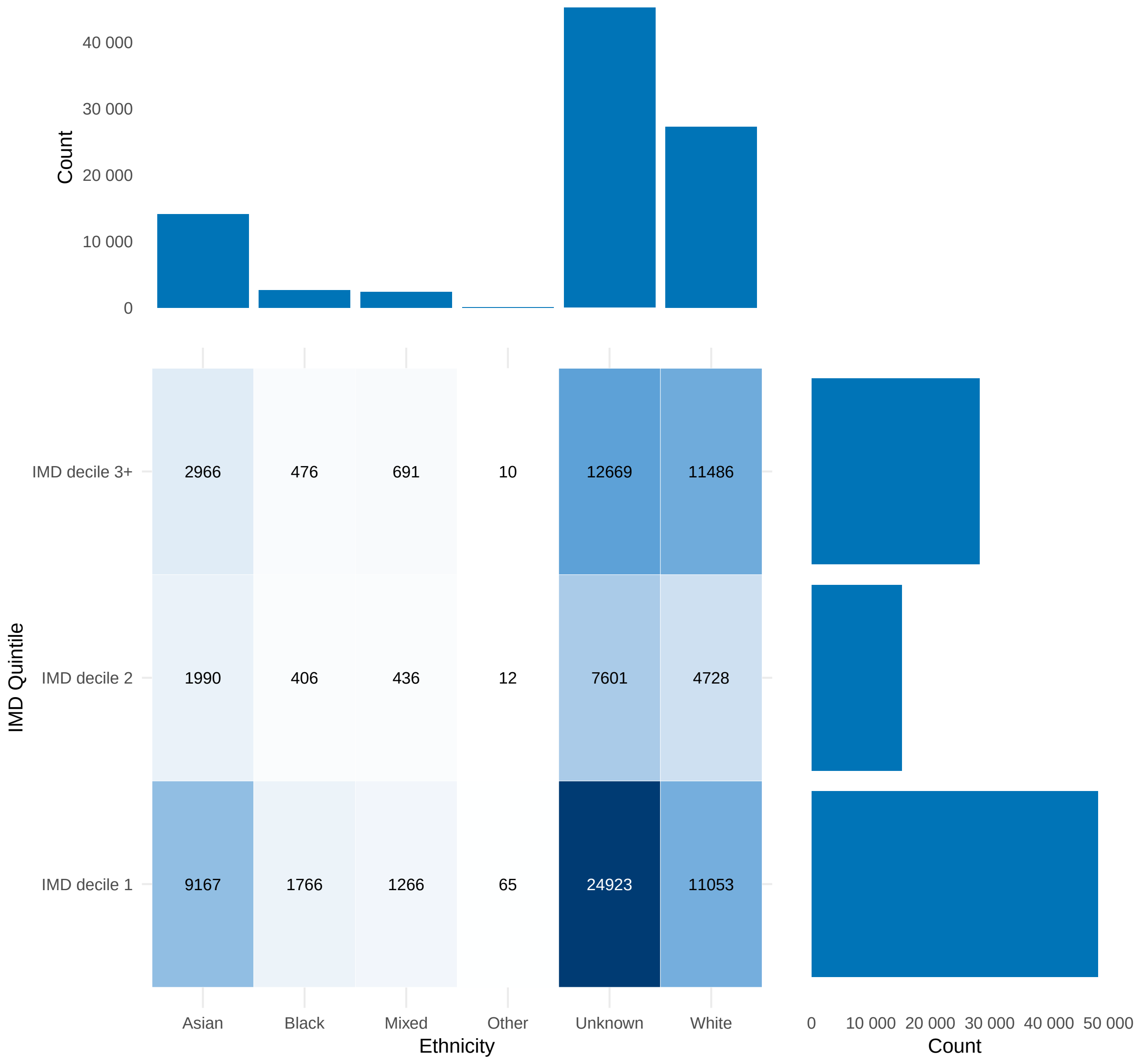

Figure S4. Distribution of HCs attendees across ethnic category and Index of Multiple Deprivation.

Table S5. The unadjusted attributable fractions of diabetes and pre-diabetes which are attributable to deprivation and to ethnicity with complete cases for deprivation or ethnicity only.

	Diabetes		Pre-Diabetes	
	**Attributable Fraction**[1]	**Excess Outcome**[1,2]	**Attributable Fraction**[1]	**Excess Outcome**[1,2]
**Socioeconomic deprivation**				
IMD quintile 3+	Reference	Reference	Reference	Reference
IMD quintile 1	65.42% (65.34% to 65.50%)	1535 (1534 to 1537)	42.66% (42.45% to 42.86%)	3111 (3096 to 3126)
IMD quintile 2	1.36% (-5.21% to 7.52%)	3 (-10 to 14)	-3.86% (-8.68% to 0.74%)	-33 (-75 to 6)
**Ethnicity**				
White	Reference	Reference	Reference	Reference
Asian	76.30% (76.18% to 76.42%)	526 (526 to 527)	65.98% (65.77% to 66.18%)	1403 (1398 to 1407)
Black	65.09% (63.50% to 66.61%)	55 (53 to 56)	67.94% (67.00% to 68.85%)	298 (294 to 302)
Mixed	61.09% (59.06% to 63.01%)	46 (45 to 48)	58.16% (56.27% to 59.97%)	177 (171 to 182)

[1] Confidence Intervals are in parentheses.

[2] Excess outcome refers to the number of outcomes in the data set that would have been avoided.

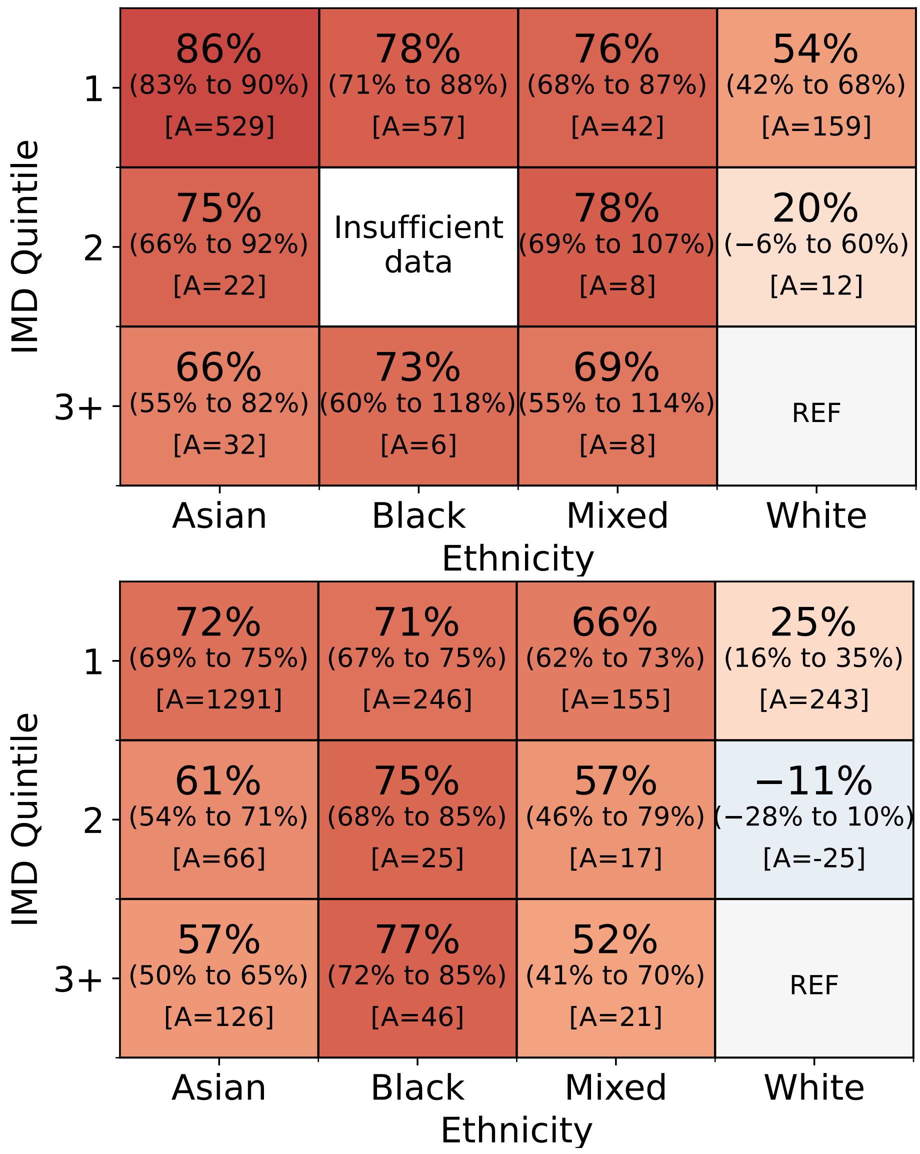

Figure S5. The unadjusted group-specific attributable fractions for diabetes (Top) and pre-diabetes (Bottom) according to socioeconomic deprivation and ethnicity with complete cases of deprivation and ethnicity only. 95% confidence intervals are in parentheses. A is the number of excess outcomes in the data set that would have been avoided. Darker colours indicate higher group attributable fraction.

7 DIAGNOSTIC TEST

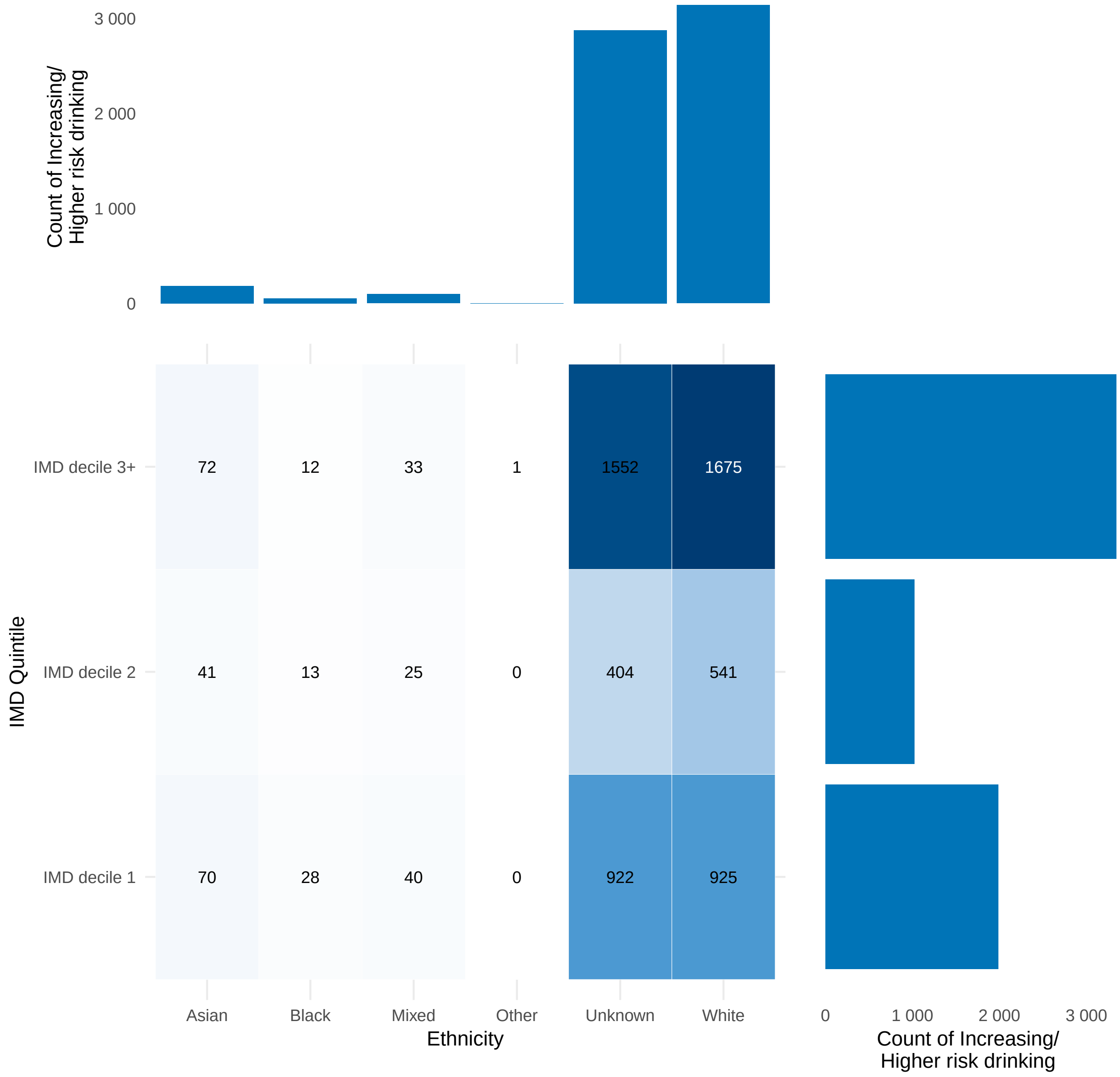

Figure S6. Distribution of HCs attendees who were categorised into ”Higher risk drinking” and ”Increasing risk drinking” across ethnic category and Index of Multiple Deprivation.

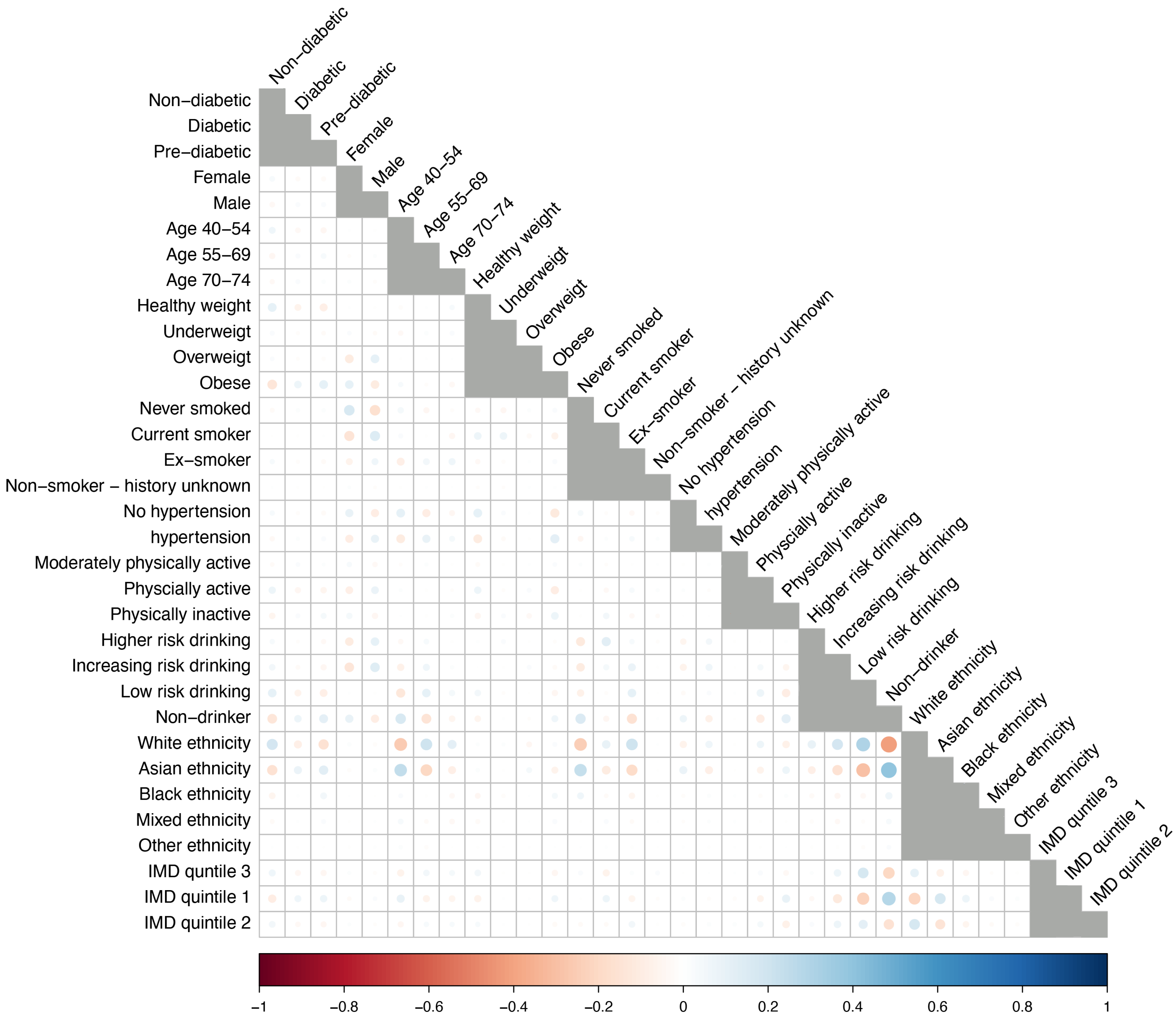

Figure S7. Correlation Matrix among outcomes and explanatory variables. Red circles indicate a negative correlation; blue circles indicate a positive correlation; blanks indicate no correlation: and grey indicate the diagonal.

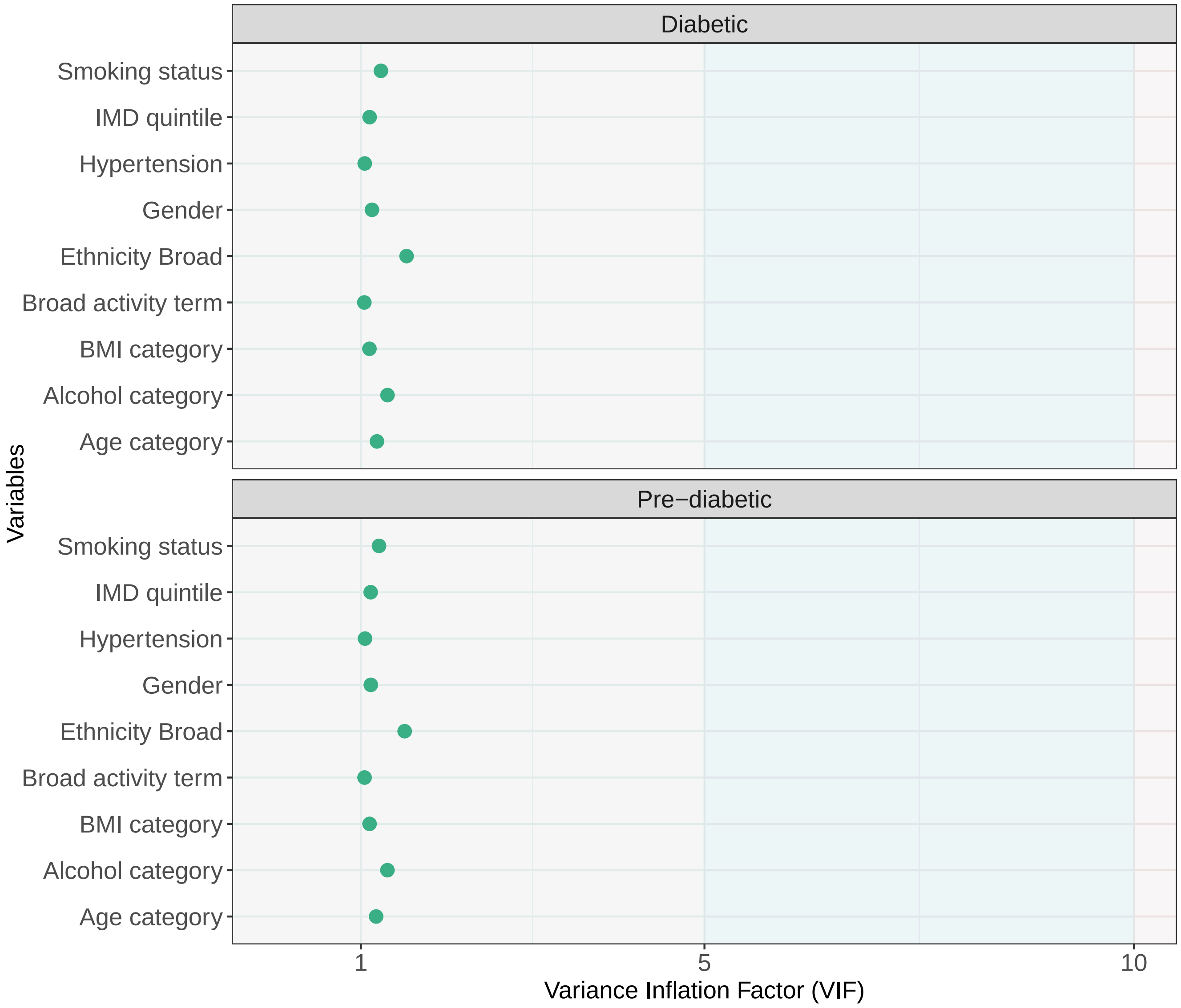

Figure S8. Variance Inflation factor (VIF) measures how much the variance of the estimated coefficients is inflated due to multicollinearity. It serves as an indicator of the extent to which independent variables in a regression model are correlated with one another.VIF values between 1 and 5 range are considered low, signifying an acceptable level of multicollinearity. VIF values between 5 and 10 are considered moderate, indicating moderate multicollinearity, while VIF values exceeding 10 are considered high, indicating severe multicollinearity.

8 COMPLETE CASE ANALYSIS

Table S6. Regression results of Complete Case Analysis

Variable	Diabetic POR[1,2]	SE[2]	Pre-diabetic POR[1,2]	SE[2]
Gender				
Female	Ref.	—	Ref.	—
Male	1.63***	0.080	1.36***	0.044
Age category				
40-54	Ref.	—	Ref.	—
55-69	2.74***	0.082	2.05***	0.045
70-74	5.06***	0.166	3.14***	0.100
BMI category				
Normal	Ref.	—	Ref.	—
Underweight	0.53	0.592	0.80	0.228
Overweight	2.29***	0.121	1.66***	0.058
Obese	5.78***	0.118	3.19***	0.058
Ethnicity Broad				
White	Ref.	—	Ref.	—
Asian	4.50***	0.100	3.60***	0.055
Black	2.13***	0.172	3.30***	0.081
Mixed	2.48***	0.161	2.68***	0.084
Other	1.57	1.02	1.99	0.483
Smoking status				
Never smoked	Ref.	—	Ref.	—
Current smoker	1.53***	0.104	1.55***	0.057
Ex-smoker	1.18	0.106	1.18**	0.058
Non-smoker - history unknown	0.98	0.226	1.07	0.121
Broad activity term				
Physically active	Ref.	—	Ref.	—
Moderately physically active	1.69***	0.103	1.24***	0.051
Physically inactive	2.02***	0.117	1.41***	0.062
Hypertension				
Normal	Ref.	—	Ref.	—
Hypertension	1.03	0.090	1.08	0.050
IMD quintile				
IMD quintile 3+	Ref.	—	Ref.	—
IMD quintile 1	1.95***	0.139	1.23**	0.064
IMD quintile 2	1.52*	0.171	0.95	0.083
Alcohol category				
Non-drinker	Ref.	—	Ref.	—
Higher risk drinking	0.59	0.318	0.38***	0.215
Increasing risk drinking	0.55**	0.197	0.54***	0.108
Low risk drinking	0.59***	0.103	0.72***	0.053

[1] *** $p < 0.001$, ** $p < 0.01$, *$p < 0.05$
[2] POR = prevalence odds ratio, SE = Standard Error
Observations = 30380

REFERENCES

Department of Health and Social Care (2021). *Delivering Better Oral Health: An Evidence-Based Toolkit for Prevention (Chapter 12: Alcohol)*. Tech. rep., United Kingdom Government. Accessed: 2023-05-24

NHS (2023). *Calculating Alcohol Units*. Tech. rep., NHS. Accessed: 2023-04-20.
